# Geoexploration of radioelement's datasets in a flood plain of crystalline bedrock

**DOI:** 10.1016/j.dib.2017.10.046

**Published:** 2017-10-24

**Authors:** Theophilus A. Adagunodo, Lukman A. Sunmonu, Moruffdeen A. Adabanija, Esivue A. Suleiman, Oluwole A. Odetunmibi

**Affiliations:** a Department of Physics, Covenant University, Ota, Nigeria; b Department of Pure and Applied Physics, Ladoke Akintola University of Technology, Ogbomoso, Nigeria; c Department of Earth Sciences, Ladoke Akintola University of Technology, Ogbomoso, Nigeria; d Department of Mathematics, Covenant University, Ota, Nigeria

**Keywords:** Radioelement, Flood plain, Crystalline bedrock, Statistical analysis, Regolith, Dose rate

## Abstract

The data in this article contains statistical analysis of radioelement in Odo-Oba flood plain of crystalline bedrock, Southwestern Nigeria. The data were acquired along twenty-two traverses. The length of each traverse is a function of its accessibility in the study area. The traverses covered the area used for agricultural practices and the area where these farm products are being sold to either the retailers or the consumers. Descriptive and multivariate statistical analyses were used to explore the measured emitted gamma radiation in Odo-Oba flood plain. The dataset can provide insights into the risks involved in exposure to outdoor radiation in a commercial centre when the average outdoor gamma radiation levels are compared to the global threshold values from the regulatory bodies such as World Health Organization, National Research Council, United States Environmental Protection Agency, Federal Environmental Protection Agency, International Commission on Radiological Protection, the United Nations Scientific Committee on the Effects of Atomic Radiation, and Federal Radiation Protection Service among others.

**Specifications Table**

**Table:** 

Subject area	*Earth Sciences*
More specific subject area	*Environmental Science*
Type of data	*Table and figure*
How data was acquired	*Gamma ray scintillometer (model GR 101A)*
Data format	*Raw and analyzed*
Experimental factors	*Radiometric measurements of radiation from a flood plain regolith was taken along traverses*
Experimental features	*Determination of gamma radiation level*
Data source location	*Odo-Oba flood plain in crystalline bedrock of Southwestern Nigeria*
Data accessibility	*All the data are in this article*

**Value of the data**

• The method can be reproduced in an area with increase in economic activities.
• The dataset can provide insights to the risks involved in exposure to outdoor radiation in a commercial centre.
• For educational purposes, radiation hazard studies in a jam-packed environment. Recent articles equivalent to the dataset presented here can be found in [1], [2], [3], [4].
• The method can be extended to other river banks where agricultural practices are done in order to know the gamma radiation level in such area.

## 1 Data

The data in this article contains the radiometric measurement of emitted gamma radiometric measurement of emitted gamma radiation from the regolith of odo-oba flood plain. The data acquired along twenty-two (22) traverses were presented in Table 1. The length of each traverse is a function of its accessibility. Over exposure to background radiation has been related to some serious health challenges which include: Chronic lung diseases, mouth necrosis, anemia, acute leucopoenia, teeth fracture, cataract, cancer, hepatic failure and leukemia. These diseases are triggered by γ-radiation, which is efficient to propagate through long distances in air in order to affect humans [5]. The descriptive statistics, Analysis of Variance (ANOVA), and multiple comparisons involving Tukey's Honest Significant Difference (HSD) test were used for the exploration of the dataset. Each statistical result is presented in the subsequent Section. The analysis can be reproduced in an area with increase in economic activities and the dosimetric quantities can be compared with the global threshold value.

**Table 1:** Gamma radiation count along each traverse (count per second).

SS	T1	T2	T3	T4	T5	T6	T7	T8	T9	T10	T11	T12	T13	T14	T15	T16	T17	T18	T19	T20	T21	T22
0	11	10.1	9.1	8.0	10.0	8.0	7.0	10	10	12	7	9	14	13	14	18	24	22	26	13	21	24
10	10.0	9.5	10.0	9.1	8.9	10.1	10	8	12	10	8	10	16.1	15	15	17	20	25	27	13	19	16
20	8.1	10.0	8.1	10.1	9.0	10.0	6	10	8	11	14	11	16	14.1	13	18	17	16	20	14	20	20
30	11.2	8.1	6.0	8.0	11.0	8.0	4	9	11	9.8	9	12.1	15	20.2	13	16	17	18	24	16	16	20
40	10.1	9.0	8.1	10.1	10.0	7.0	9	14	12	10.1	0	9	14	16	16	14	18	16	22	18	18	16
50	8.2	–	–	10.7	11.0	10.0	10	11	12.1	9	10.2	8	13	13	14	16	19	16	–	20	22	20
60	10.0	–	–	12.0	9.1	14.0	8	13	–	–	12	7	13	14	13	16	18	18	–	–	–	26
70	–	–	–	–	11.1	9.0	–	11	–	–	11	9	–	14	15	13	18	12	–	–	–	23
80	–	–	–	–	10.1	11.0	–	14	–	–	9	7	–	15	15	16	15	18	–	–	–	22
90	–	–	–	–	–	12.0	–	–	–	–	8	8	–	16	20	15	13.2	19	–	–	–	18
100	–	–	–	–	–	–	–	–	–	–	7	–	–	15	14	18	19	18	–	–	–	22
110	–	–	–	–	–	–	–	–	–	–	6	–	–	–	16	16	17	–	–	–	–	20
120	–	–	–	–	–	–	–	–	–	–	5	–	–	–	13	18.1	16	–	–	–	–	20
130	–	–	–	–	–	–	–	–	–	–	–	–	–	–	15	18	17	–	–	–	–	19
140	–	–	–	–	–	–	–	–	–	–	–	–	–	–	18	17	18	–	–	–	–	20
150	–	–	–	–	–	–	–	–	–	–	–	–	–	–	18	18	19	–	–	–	–	18
160	–	–	–	–	–	–	–	–	–	–	–	–	–	–	15	16	20	–	–	–	–	18
170	–	–	–	–	–	–	–	–	–	–	–	–	–	–	13	15	–	–	–	–	–	–
180	–	–	–	–	–	–	–	–	–	–	–	–	–	–	18	16	–	–	–	–	–	–
190	–	–	–	–	–	–	–	–	–	–	–	–	–	–	15	–	–	–	–	–	–	–
200	–	–	–	–	–	–	–	–	–	–	–	–	–	–	18	–	–	–	–	–	–	–

*Note:* SS denotes Station Separations (metre); T1…T22 denotes Traverse 1 … Traverse 22 (count per second); - denotes End of Traverse.

## 2 Experimental design, materials and methods

In retrospect, some datasets have been analyzed in Odo-Oba, Southwestern Nigeria in the last one decade. The studies include radiometric signatures analysis, morphometry assessment using geophraphic information system data, internal geometry assessment using electrical resistivity tomography technique, evaluation of heavy metals in soil samples, and water assessment of Odo-Oba, Nigeira [6], [7], [8], [9].

### 2.1 Study area

**Fig. 1:**
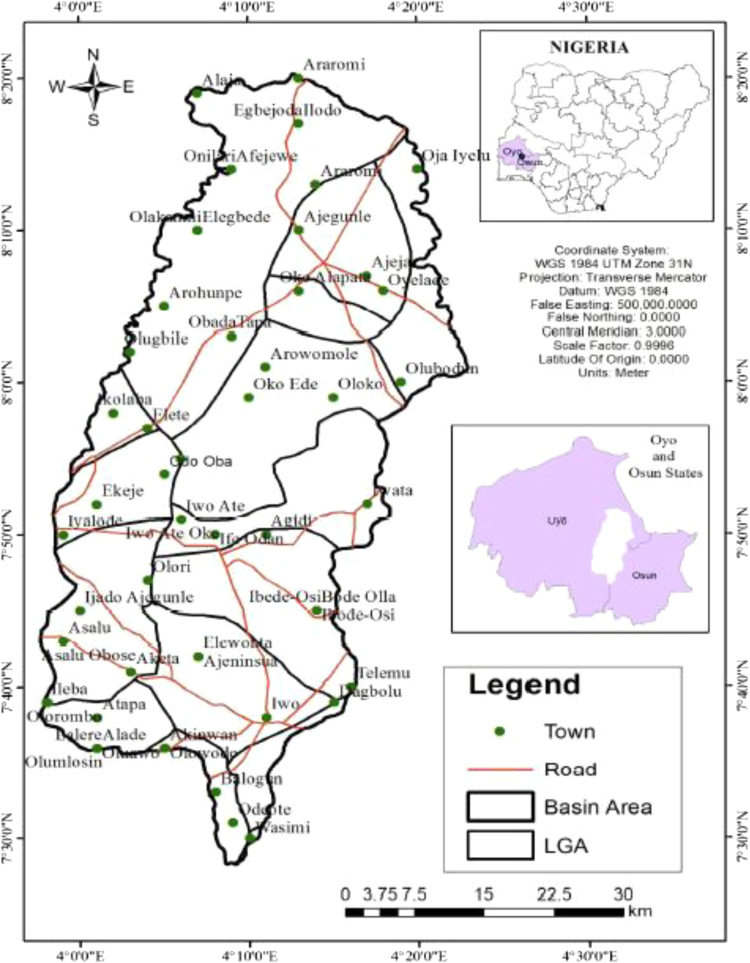
Map of Oba river basin (Adapted from [7]).

Oba river basin is located between Oyo and Osun states, southwestern Nigeria (Fig. 1). The basin is bounded with the coordinates of latitude 7° 28′ 25.9′′ to 8° 18′ 51.3′′ north and latitude 4° 8′ 44.3′′ to 4° 13′ 14.1′′ east respectively [7]. As reported by [8] that, “for several years, most of the materials being carried by the river several kilometers are deposited around Oba village along Oyo-Ogbomoso road probably because of the relatively planar surface of the area, this action has resulted to alluvial plain (quaternary sediments) which are likely to be made up of different materials”. Oba river basin is located in a warm tropic region of the rain forest of southwestern Nigeria. The climate in the Northern part of the basin, via Ogbomoso is of high temperature. Moderate to heavy seasonal rainfall is experienced from March to July with an average annual rainfall of 1247 mm. The Relative Humidity (RH) is usually high in the morning and decreases towards the afternoon. This process occurs throughout the year. Annually, high RH is experienced from July to September while low RH occurs from December to February. During the dry season, the tropical continental air mass blows across the study area. The wind picks little or no moisture which is further influenced by the tropical air mass during the rainy season.

**Fig. 2:**
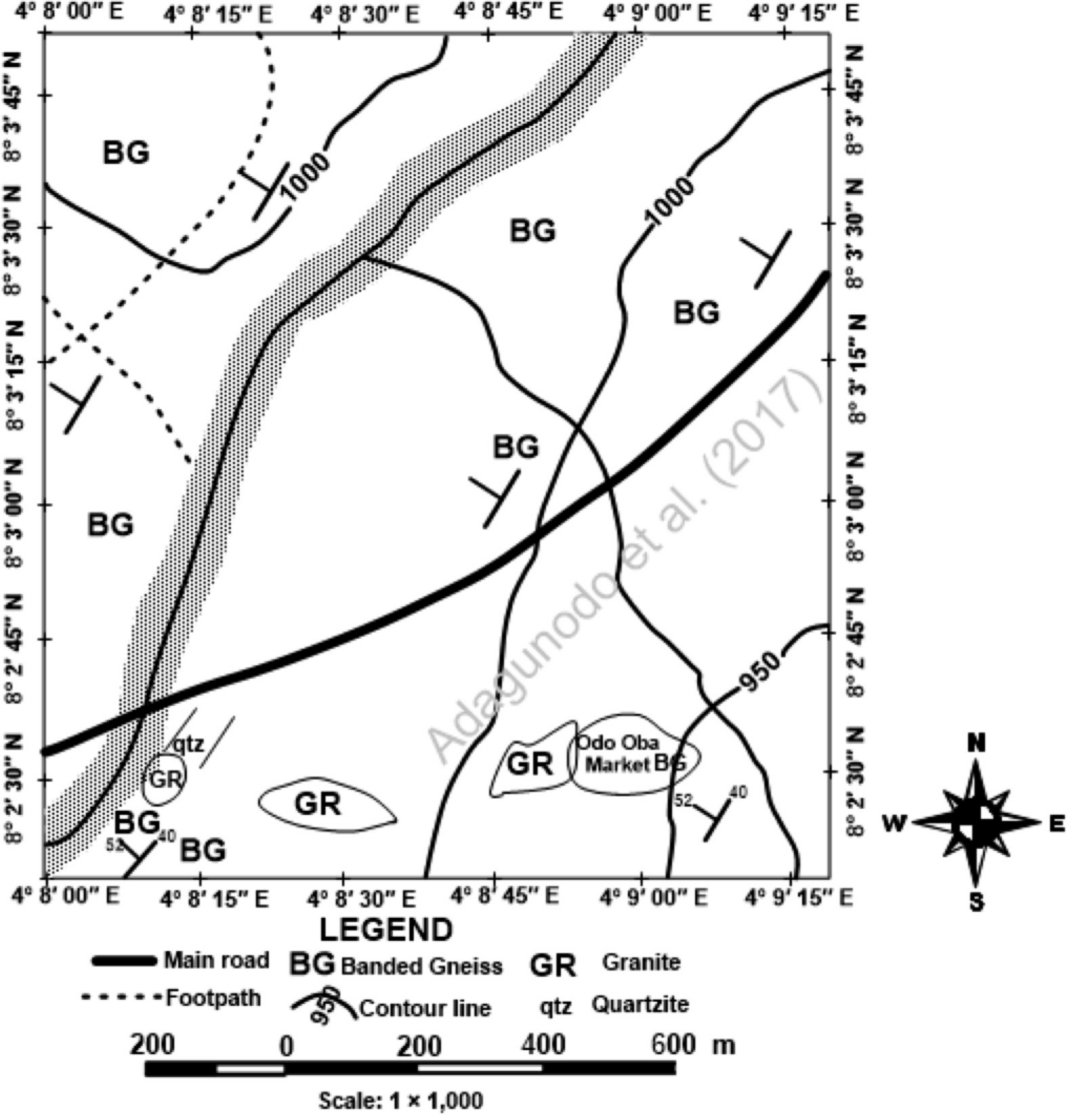
Odo-Oba geological map (adapted from [8]).

Geologically, Odo-Oba is underlain majorly by banded gneiss and quartzite with minor distribution of pegmatite in the study area (Fig. 2). The regolith revealed a shallow weathered profile reposing directly on the basement rock. This is confirmed from the depth of hand-dug wells in the study area which are virtually 5 m deep. The residents along the whole length of Oba River are mostly into farming and fishing. The major crops being cultivated are maize, okra, vegetables, water melon and garden egg.

### 2.2 Materials and methods

Gamma ray scintillometer (model GR 101A) was used to map the gamma radiation variations on Odo-Oba flood plain along twenty-two (22) traverses. The interstation distance of 10 m interval was adopted. Gamma ray Scintillometer uses the Geiger-Muller (G-M) counter's principle. Pulse measurement was done in counts per second (cps) regardless of the energy being corresponded to its radiation interaction. The measurements were done at full-scale range of 0.1k and audio signal of 75%. The measured gamma radiation count was converted to the dose rate using Eq. (1) and presented in Table 2.

(1) A=1.9514B

where A is the gamma count rate in counts per second (cps) and B is the dose rate in $\mu Svh^{-1}$. The model is based on calibration of the scintillometer at the Federal Radiation Protection Service, University of Ibadan, Ibadan, Nigeria. However, the respective mean of these count rates and dose rates can be compared with the global threshold values.

**Table 2:** Dose rate $\left(\mu Svh^{-1}\right)$.

**SS**	**T1**	**T2**	**T3**	**T4**	**T5**	**T6**	**T7**	**T8**	**T9**	**T10**	**T11**	**T12**	**T13**	**T14**	**T15**	**T16**	**T17**	**T18**	**T19**	**T20**	**T21**	**T22**
0	5.64	5.18	4.66	4.10	5.12	4.10	3.59	5.12	5.12	6.15	3.59	4.61	7.17	6.66	7.17	9.22	12.30	11.27	13.32	6.66	10.76	12.30
10	5.12	4.87	5.12	4.66	4.56	5.18	5.12	4.10	6.15	5.12	4.10	5.12	8.25	7.69	7.69	8.71	10.25	12.81	13.84	6.66	9.74	8.20
20	4.15	5.12	4.15	5.12	4.61	5.12	3.07	5.12	4.10	5.64	7.17	5.64	8.20	7.23	6.66	9.22	8.71	8.20	10.25	7.17	10.25	10.25
30	5.74	4.15	3.07	4.10	5.64	4.10	2.05	4.61	5.64	5.02	4.61	6.20	7.69	10.35	6.66	8.20	8.71	9.22	12.30	8.20	8.20	10.25
40	5.18	4.61	4.15	5.18	5.12	3.59	4.61	7.17	6.15	5.18	0	4.61	7.17	8.20	8.20	7.17	9.22	8.20	11.27	9.22	9.22	8.20
50	4.20	–	–	5.48	5.64	5.12	5.12	5.64	6.20	4.61	5.23	4.10	6.66	6.66	7.17	8.20	9.74	8.20	–	10.25	11.27	10.25
60	5.12	–	–	6.15	4.66	7.17	4.10	6.66	–	–	6.15	3.59	6.66	7.17	6.66	8.20	9.22	9.22	–	–	–	13.32
70	–	–	–	–	5.69	4.61	–	5.64	–	–	5.64	4.61	–	7.17	7.69	6.66	9.22	6.15	–	–	–	11.79
80	–	–	–	–	5.18	5.64	–	7.17	–	–	4.61	3.59	–	7.69	7.69	8.20	7.69	9.22	–	–	–	11.27
90	–	–	–	–	–	6.15	–	–	–	–	4.10	4.10	–	8.20	10.25	7.69	6.76	9.74	–	–	–	9.22
100	–	–	–	–	–	–	–	–	–	–	3.59	–	–	7.69	7.17	9.22	9.74	9.22	–	–	–	11.27
110	–	–	–	–	–	–	–	–	–	–	3.07	–	–	–	8.20	8.20	8.71	–	–	–	–	10.25
120	–	–	–	–	–	–	–	–	–	–	2.56	–	–	–	6.66	9.28	8.20	–	–	–	–	10.25
130	–	–	–	–	–	–	–	–	–	–	–	–	–	–	7.69	9.22	8.71	–	–	–	–	9.74
140	–	–	–	–	–	–	–	–	–	–	–	–	–	–	9.22	8.71	9.22	–	–	–	–	10.25
150	–	–	–	–	–	–	–	–	–	–	–	–	–	–	9.22	9.22	9.74	–	–	–	–	9.22
160	–	–	–	–	–	–	–	–	–	–	–	–	–	–	7.69	8.20	10.25	–	–	–	–	9.22
170	–	–	–	–	–	–	–	–	–	–	–	–	–	–	6.66	7.69	–	–	–	–	–	–
180	–	–	–	–	–	–	–	–	–	–	–	–	–	–	9.22	8.20	–	–	–	–	–	–
190	–	–	–	–	–	–	–	–	–	–	–	–	–	–	7.69	–	–	–	–	–	–	–
200	–	–	–	–	–	–	–	–	–	–	–	–	–	–	9.22	–	–	–	–	–	–	–

*Note:* SS denotes Station Separations (metre); T1…T22 denotes Traverse 1 … Traverse 22 (count per second); - denotes End of Traverse.

### 2.3 Statistical analysis

**Fig. 3:**
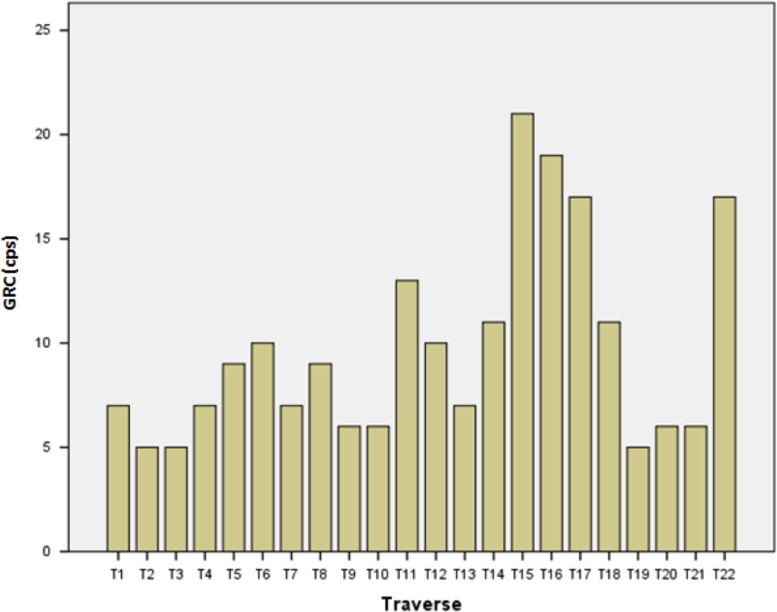
GRC distribution in the study area.

**Fig. 4:**
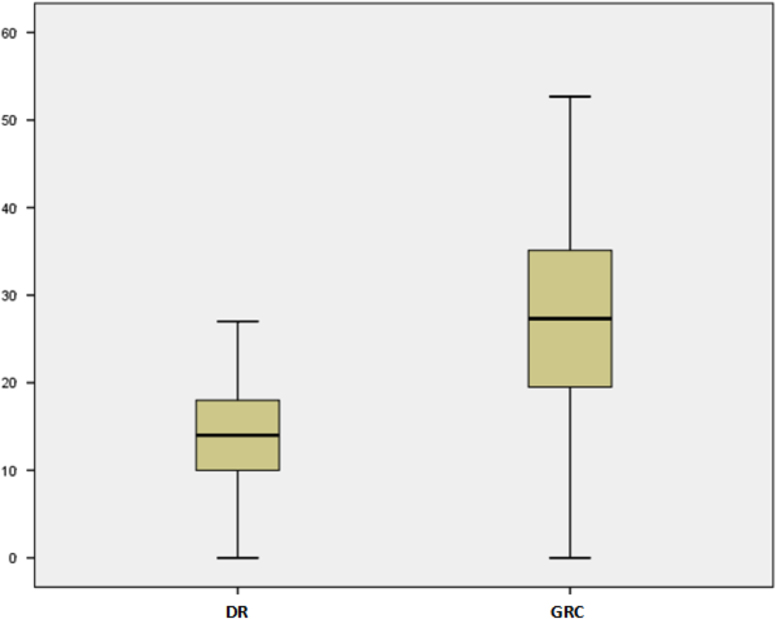
Box-plot comparing the distribution of the DR against the GRC.

Table 3 gives the descriptive statistics for both Dose Rate (DR) and Gamma Radiation Count (GRC) with positive skewness and negative kurtosis respectively, implying the asymmetric and light-tailed distribution of the data, while Fig. 3 shows the chart representation of the GRC in the study area. Fig. 3 reveals that T15 possesses the highest count and T2, T3, and T19 produced the lowest count in the study area. From the boxplot (Fig. 4), which helps describe the distribution of the data, there are no outliers in the reading from both the GRC and DR. The statistical attributes (minimum, 1st quartile, median, 3rd quartile and maximum) of GRC are almost double that of DR.

**Table 3:** Descriptive statistics for gamma radiation count and dose rate.

Description	N	Min.	Max.	Mean	Mean std. error	Std. deviation	Skewness	Skewness std. error	Kurtosis	Kurtosis std. error
DR	214	0.0	27.00	13.85	0.33	4.76	0.256	0.166	−0.293	0.331
GRC	214	0.0	52.69	27.03	0.64	9.30	0.256	0.166	−0.293	0.331
Valid N (listwise)	214									

**Fig. 5:**
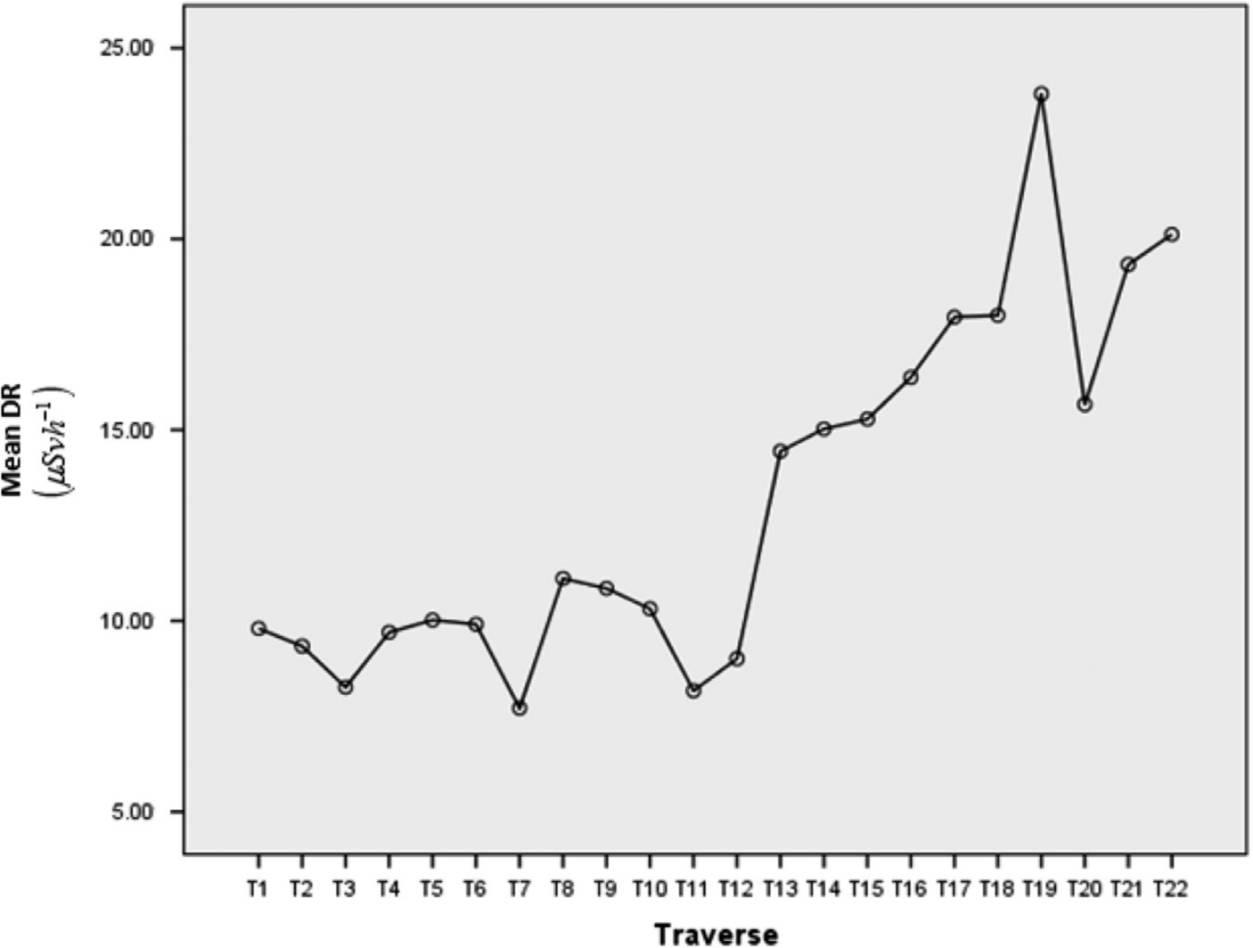
Mean dose rate distribution in the study area.

**Fig. 6:**
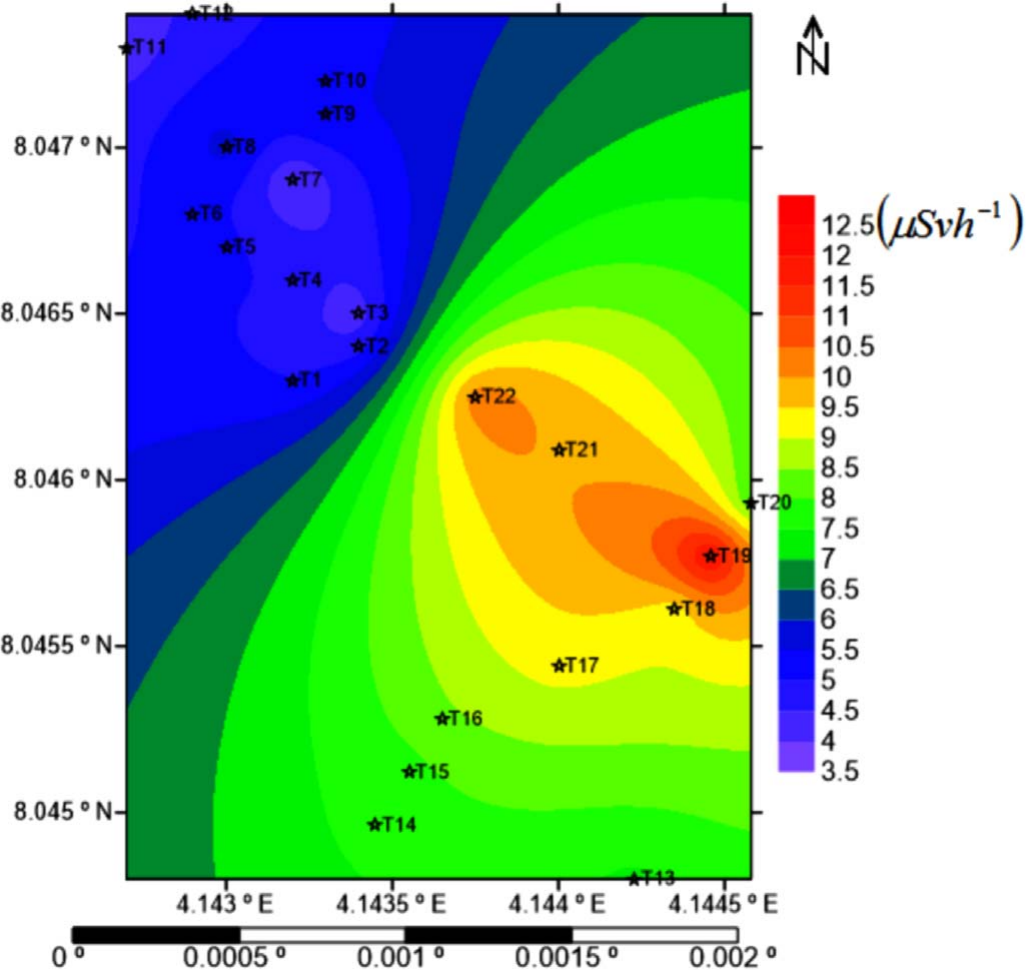
The average dose rate distributions in the study area.

The descriptive statistics (N, mean, standard deviation, etc.) of the traverse (independent variable) and DR (dependent variable) are presented in the Table 4, with T15 having the highest mean and standard deviation score (Mean (M)=15.29, Standard Deviation (SD)=2.05) while T2 (M=9.34,SD=0.82), T3 (M=8.26,SD=1.49), and T19 (M=23.8,SD=2.86) showed the lowest score respectively. Meanwhile, Table 5 gives the Levene's test for homogeneity of variances, which tests whether the variance in scores is the same for each of the twenty-two (22) groups of the Traverse. From this table, it was deduced that the assumption of homogeneity of variance was not violated since the significant value (Sig.) was greater than 0.05(=0.212) with Levene Statistic as 1.253. The Analysis of Variance (ANOVA) Table (that is, Table 6) was implemented to explore the impact of the Traverse on the DR. The one way ANOVA statistic also detected whether the differences in the mean scores of the Traverse groups are statistically significant, with a claim (null hypothesis) that means between the 22 groups are equal. From Table 6, it can be deduced that at a 5% level of significance, the mean score between groups are significant among some of the groups with a p-value (Sig.) lower than the level of significance: F(21,192)=38.925,p=0.00<0.05. Though the ANOVA test detected significant differences between the groups (Traverse) under investigation, a Post-Hoc test (Tukey's HSD test) for multiple comparison was further conducted to determine the exact Traverses where significant differences lie. However for clarity purpose, a summary of the features of the Traverses that were significantly different were given in Table 7 and Table 8. Table 8 presents a matrix representation of significant and non-significant Traverses with the cells having an asterisk sign (*) indicating statistically significant Traverses and empty cells showing no significant differences in mean scores between compare groups. For instance, in illustrating Table 7, T1 is significant at 5% level from T13 to T22, T2 is significant from T13 to T22, etc., while for Table 8, T1 is significant to T13 and non-significant to T2, T1 is significant to T14, and so on. From the Mean dose rate (Fig. 5), which is an easy way to compare the mean scores of different groups, it can be observed that the Traverse with the lowest mean DR is T7 and that with the highest mean DR is T19. These values can be compared with the world threshold value to determine whether the study area is safe or not. However, Fig. 6 shows the 2-D plot of the average dose rate distributions in the study area. The map shows that high gamma radiation count trend in southeastern part while the northwestern region is contained with low gamma radiation count.

**Table 4:** Descriptive statistics of the impact of the traverse on the dose rate.

Traverse	N	Mean	Std. deviation	Std. error	95% confidence interval for mean		Minimum	Maximum	Between-Component Variance
					Lower bound	Upper bound			
T1	7	9.80	1.23	0.46	8.67	10.93	8.10	11.20	
T2	5	9.34	0.82	0.37	8.32	10.36	8.10	10.10	
T3	5	8.26	1.49	0.67	6.41	10.11	6.00	10.00	
T4	7	9.70	1.45	0.55	8.36	11.04	8.00	12.00	
T5	9	10.02	0.88	0.29	9.34	10.70	8.90	11.10	
T6	10	9.91	2.08	0.66	8.42	11.40	7.00	14.00	
T7	7	7.71	2.21	0.84	5.67	9.76	4.00	10.00	
T8	9	11.11	2.15	0.72	9.46	12.76	8.00	14.00	
T9	6	10.85	1.62	0.66	9.15	12.55	8.00	12.10	
T10	6	10.32	1.04	0.43	9.22	11.41	9.00	12.00	
T11	13	8.17	3.49	0.97	6.06	10.28	0.00	14.00	
T12	10	9.01	1.65	0.52	7.83	10.19	7.00	12.10	
T13	7	14.44	1.29	0.49	13.25	15.64	13.00	16.10	
T14	11	15.03	2.00	0.60	13.69	16.37	13.00	20.20	
T15	21	15.29	2.05	0.45	14.35	16.22	13.00	20.00	
T16	19	16.37	1.47	0.34	15.67	17.08	13.00	18.10	
T17	17	17.95	2.33	0.57	16.75	19.15	13.20	24.00	
T18	11	18.00	3.38	1.02	15.73	20.27	12.00	25.00	
T19	5	23.80	2.86	1.28	20.24	27.36	20.00	27.00	
T20	6	15.67	2.88	1.17	12.65	18.68	13.00	20.00	
T21	6	19.33	2.16	0.88	17.07	21.60	16.00	22.00	
T22	17	20.12	2.67	0.65	18.75	21.49	16.00	26.00	
Total	214	13.85	4.76	0.33	13.21	14.49	0.00	27.00	
Fixed effects model			2.19	0.15	13.55	14.14			
Random effects				1.04	11.69	16.01			18.88

**Table 5:** Test for homogeneity of variances on the estimated dose rate.

Levene's statistic	df1	df2	Significant
1.253	21	192	0.212

**Table 6:** ANOVA of the traverses on the dose rate.

Category	Sum of Squares	Df	Mean square	F	Significant
Between groups	3914.814	21	186.420	38.925	0.000
Within groups	919.521	192	4.789		
Total	4834.335	213

**Table 7:** Summary of the results from the multiple comparison table.

Traverse	Significant traverses at α=0.05	Traverse	Significant traverses at α=0.05	Traverse	Significant traverses at α=0.05
T1	T13–T22	T9	T14–T22	T17	T1–T12, T15, T19
T2	T13–T22	T10	T14–T22	T18	T1–T12, T19
T3	T13–T22	T11	T14–T22	T19	T1–T20
T4	T13–T22	T12	T14–T22	T20	T1–T12, T19, T22
T5	T13–T22	T13	T1–T7, T11–T12, T19, T21–T22	T21	T1–T15
T6	T13–T22	T14	T1–T12, T19, T21–T22	T22	T1–T16, T20
T7	T13–T22	T15	T1–T12, T17, T19, T21–T22		
T8	T14–T22	T16	T1–T12, T19, T22

**Table 8:** Matrix representation of the traverse categories that are significant at 5% level.

*	T1	T2	T3	T4	T5	T6	T7	T8	T9	T10	T11	T12	T13	T14	T15	T16	T17	T18	T19	T20	T21	T22
T1													*	*	*	*	*	*	*	*	*	*
T2													*	*	*	*	*	*	*	*	*	*
T3													*	*	*	*	*	*	*	*	*	*
T4													*	*	*	*	*	*	*	*	*	*
T5													*	*	*	*	*	*	*	*	*	*
T6													*	*	*	*	*	*	*	*	*	*
T7													*	*	*	*	*	*	*	*	*	*
T8												*	*	*	*	*	*	*	*	*	*	*
T9												*	*	*	*	*	*	*	*	*	*	*
T10												*	*	*	*	*	*	*	*	*	*	*
T11													*	*	*	*	*	*	*	*	*	*
T12													*	*	*	*	*	*	*	*	*	*
T13	*	*	*	*	*	*	*				*	*							*		*	*
T14	*	*	*	*	*	*	*	*	*	*	*	*							*		*	*
T15	*	*	*	*	*	*	*	*	*	*	*	*					*		*		*	*
T16	*	*	*	*	*	*	*	*	*	*	*	*							*			*
T17	*	*	*	*	*	*	*	*	*	*	*	*			*				*			
T18	*	*	*	*	*	*	*	*	*	*	*	*							*			
T19	*	*	*	*	*	*	*	*	*	*	*	*	*	*	*	*	*	*	*	*		
T20	*	*	*	*	*	*	*	*	*	*	*	*							*			*
T21	*	*	*	*	*	*	*	*	*	*	*	*	*	*	*							
T22	*	*	*	*	*	*	*	*	*	*	*	*	*	*	*	*				*		

^*^ Significant categories among traverse for the dose rate of the study area.
