# Supplementary material for: Geoexploration of radioelement's datasets in a flood plain of crystalline bedrock
Source: Data Brief. 2017 Oct 24;15:809–20. doi: 10.1016/j.dib.2017.10.046 (PMC5676079; doi:10.1016/j.dib.2017.10.046)
Supplement: Supplementary file 1 — Supplementary material [file mmc1.docx]

The authors declare no conflict of interest in this article.

'Conflicts of interest: none'.
